# Isolation of Bacteriophages and Their Lytic Profile From River and Waste Water Samples in South Western Uganda

**DOI:** 10.7759/cureus.102524

**Published:** 2026-01-28

**Authors:** Judith Owokuhaisa, Keneth Junior Male, Richard Ezinga, Edgar Mulogo, Moses Ntaro, Moses Mpeirwe, Joel Bazira

**Affiliations:** 1 Department of Microbiology, Mbarara University of Science and Technology, Mbarara, UGA; 2 Department of Biochemistry, Mbarara University of Science and Technology, Mbarara, UGA; 3 Department of Community Health, Mbarara University of Science and Technology, Mbarara, UGA

**Keywords:** bacteria, host range, lytic bacteriophages, river, waste water

## Abstract

Background

Bacteriophages are found almost everywhere in the environment, such as water, soil, and air, among others. They are effective against a wide range of pathogens because they are highly specific. This study aimed to identify phages from river and wastewater samples in south-western Uganda and determine their lysis profile against selected clinical bacterial isolates.

Materials and methods

Four different locations (Mbarara city and Ntungamo, Kabale, and Kisoro districts) in south-western Uganda were chosen for the collection of river and wastewater for isolation of potential phages. The culture and double agar overlay method was used to detect the lytic bacteriophages. Isolated phages were purified after diluting them in saline magnesium (SM) buffer, then centrifuged at 3,000 rpm for 10 minutes, and filtered through a 0.22 um syringe filter. After purification, the filtrate was used to determine the phage lytic activity, host range activity (using the spot method), and thermal stability (using the double agar overlay method).

Results

A total of 17 phages were isolated using *Escherichia coli* WG5 as the host. The phage concentration ranged from 5.5 x 10^6^ -7.6 x 10^11^ pfu/mL. All the isolated phages were used for host range determination on *Salmonella *spp., *Morganelli morganii, *extended-spectrum beta-lactamase (ESBL)* E. coli, Proteus mirabilis, Citrobacter freundii, Coryne bacteria *spp., and *Serratia marcescens* bacterial isolates. Of the 17 phages, ECWG5_I_MbraKat_OJ_25 and ECWG5_RKirUS_OJ_25 phages were the most effective against the tested bacterial strains, with 75% lytic ability. These two phages could lyse ESBL *E. coli*. The isolated phages were stable at 4 °C and 37 °C, and their dilution end point range was 10^-6^ to 10^-10^, suggesting that the phage stocks were still potent and able to infect bacteria at relatively high dilutions.

Conclusion

The study isolated 17 phages from wastewater and the river water, which showed lytic activity against selected clinical Gram-positive and Gram-negative bacterial isolates. This study offers preliminary insights into the environmentally isolated phages, enabling researchers to further characterize and assess their potential application in phage-based clinical interventions, particularly for the management of drug-resistant pathogenic bacteria.

## Introduction

Viruses that specifically infect bacteria (bacteriophages or phages) are ubiquitous. Phages are the most abundant viruses on earth and need a susceptible bacterial host to replicate [1]. Phage therapy is an urgently needed alternative to control bacterial pathogens [2], although the use of phage therapy needs to be well studied [3]. Understanding the phage-bacterial interactions is important for successful phage therapy development and application [4].

Phages are not able to multiply in eukaryotic cells or insert their genetic material into the genome of such cells because of their specificity to infect bacteria. They are highly specific, and each infects only a single bacterial species, and some are further specialized to target particular strains of that species [5,6]. Soils, sewage, animal secretions, and rivers, among others, are unique sources of all kinds of phages, hence offering a possibility to easily isolate them for therapeutic purposes. Additionally, a primary benefit of phages is their natural existence alongside pathogenic bacteria, particularly within sewage and wastewater systems that collect diverse environmental contaminants.

Applying phages to neutralize specific harmful bacteria offers a compelling substitute for conventional treatments [7]. Despite the growing threat of antimicrobial resistance in sub-Saharan Africa, including Uganda, and the effort given to its management, novel alternatives such as phage therapy are still in their infancy. In addition, studies on phage isolation from various environmental samples in Uganda, in particular the south western regions, as well as determining their lytic profile, are largely lacking. Therefore, we carried out a descriptive study to identify phages from river and wastewater samples in South Western Uganda and determine their lytic profile against selected clinical bacterial isolates.

## Materials and methods

Sample collection

Study samples were collected from selected areas of south-western Uganda, namely, wastewater treatment plants (WWTPs) in the districts of Kabale, Ntungamo, and Kisoro and Mbarara city. Additionally, samples were collected from rivers, Kiruruma and Rwizi, and sewage from Mbarara Regional Referral Hospital (MRRH). Samples were processed at Mbarara University of Science and Technology (MUST) Research Clinical and Microbiology laboratories from April 2025 to September 2025. The south-western region comprises 16 districts (Kabale, Mbarara, Ntungamo, Rwampara, Kiruhura, Kazo, Ibanda, Sheema, Bushenyi, Mitooma, Rubanda, Rukiga, Kisoro, Kanungu, and Isingiro). MRRH was selected because it provides a range of health services and serves a wider population of people who come from other regions in the country and beyond. It has a bed capacity of 350 and serves the catchment districts of Ibanda, Mbarara, Kazo, Bushenyi, Ntungamo, Kiruhura, Buhweju, Mitooma, Sheema, Rubirizi, Rwampara, Isingiro, and Mbarara city. The hospital releases the waste to WWTPs (stabilization ponds) directly, which joins the River Rwizi. The River Rwizi is located in south-western Uganda and traverses various districts, including Ntungamo and Sheema. It originates from the Buhweju hills and covers the catchment area of 2,521 km^2^ [8]. The river discharges its water into Lake Victoria through Mburo, Nakivale, Kachera, and Kijanebarola lakes [9]. River Kiruruma is a stream in Kabale district, western Uganda and has an elevation of 1,924 m. The WWTP in Kabale discharges its effluent in river Kiruruma, which is situated near Ishingiro and Kumba villages.

Study design and sampling method

A descriptive and laboratory cross-sectional study was carried out to generate preliminary data on lytic phages present in the sampling sites. A non-probability purposive sampling method was selected for its practicality and convenience in this study. Sampled sites were influent, effluent, hospital septic tank, upstream, and downstream of the WWTPs. A total of 17 samples were collected, including nine from Mbarara city - covering River Rwizi (upstream and downstream), hospital wastewater, and influent and effluent from Katete, Kakoba, and Kijungu wastewater treatment plants. Additionally, four samples were obtained from Kabale district (River Kiruruma upstream and downstream, as well as influent and effluent), two from Ntungamo district (influent and effluent), and two from Kisoro district (influent and effluent), as shown in Table 1.

**Table 1:** Sources of sewage and river water samples MRRH - Mbarara Regional Referral Hospital, WWTPs - Waste Water Treatment Plants

Sources of river and waste water samples	Sampling points	Total number of samples collected from each source
Hospital waste		
MRRH	Septic tank	1
Stabilization ponds (WWTPs)		
Mbarara (Kakoba, Kijungu, Katete)	Influent & effluent	6
Kabale (Kigongi)	Influent & effluent	2
Ntungamo (Kyamate)	Influent & effluent	2
Kisoro (Seseme)	Influent & effluent	2
River water		
River Rwizi (Mbarara)	Upstream & downstream	2
River Kiruruma (Kabale)	Upstream & downstream	2

Sample collection and transportation

After receiving approval from the National Water and Sewerage Corporation, the Ministry of Water and Environment, and MUST Research Ethics Committee, 1 L of each sample was collected from respective sample sites in a 1 L sterile bottle (Borosil R 1501, R trace Code, 2901FE025E). The samples were transported to the laboratory (MUST Clinical Research Laboratory) in an ice box and stored at 4 °C until processed.

Bacterial host strain and culture condition

The bacterial host used in this study to isolate the phages was *E. coli* (WG5 DSM 18455), which was a working stock culture supplied by the Surveillance of Emerging Pathogens and Antibiotic Resistances in Aquatic Ecosystems (SARA) project at MUST. The bacterial isolates were stored as glycerol stocks at -80 °C. The *E. coli *WG5 DSM 18455 host isolates were subcultured in Luria-Bertani (LB) broth with shaking at 100 rpm at 37 °C for 24 hours. The bacteria isolated used to determine the lytic activity of isolated phages included eight clinically isolated bacteria from pus, wound, urine, and stool. These were culture collections at the MUST Microbiology Laboratory. The bacterial isolates were stored as glycerol stocks at -20 °C and included *Salmonella typhimurium *ATCC14028 (used as a standard strain); *S. typhimurium *WG49 NCTC 12484 (widely used in research, which was sourced from the SARA project at MUST); *Morganelli morganii; *ESBL *E. coli; Proteus mirabilis; Citrobacter freundii; Corynebacterium *spp.; and *Serratia marcescens.*

Phage isolation

The double agar layer assay was performed for phage isolation according to [6,10] with a brief modification. First, the *E. coli *WG5 DSM 18455 host isolates were subcultured in LB broth with shaking at 100 rpm at 37 °C for 24 hours, until an optical density (OD) of 0.5 at 600 nm. Then, 1 mL and 2 mL of the sample (wastewater and river water, respectively) were transferred into a sterile 250 mL bottle for each of the samples (waste or river water). The volume of river water samples was doubled (we anticipated a low phage count in these samples). To reduce background flora in waste samples (influent), 50 µL of nalidixic acid was added. Then, 1 mL of *E. coli *WG5DSM18455 (host bacteria) grown in LB broth was added and mixed well. The mixture was left at ambient temperature for 10 minutes to allow the phages to adsorb to the host bacteria. After 10 minutes, 5 mL of molten LB agar (0.7%W/V) supplemented with 50 µL of 2 mM calcium chloride was added, mixed well, and poured over LB agar plates. The plates were allowed to dry for 10 minutes at room temperature and incubated upside down at 37 °C for 24 hours. Each sample was tested in triplicate. The plates were observed for plaque formation over the plate surface. Plaques were counted and recorded as plaque forming unit per milliliter (pfu/mL).

Phage purification

Plates with clear plaques were selected and washed with 10 mL of saline magnesium (SM) buffer (100 mM NaCL, 8 mM MgSO_4_, and 50 mM Tris-HCL (pH 7.4) and 0.01% of gelatin) and left at ambient temperature at 4 °C overnight. A sterile scrapper was used to scrape off the top soft agar layer, which was collected together with the SM buffer into 15 mL Falcon tubes. The mixture was centrifuged at 3,000 rpm for 10 minutes to remove the agar and bacterial cells. The supernatant was filtered through a 0.22 µm syringe filter to remove the remaining bacterial cells and debris. The phage lysate was stored at 4 °C until processing [11].

Phage titer determination

The pfu/mL, a measure of the phage concentration, was used to calculate the phage titer by counting the number of plaques that developed on cultured LB agar plates according to Hasanain et al. [6] and Khorshidtalab et al. [12]. After serial dilution (1:100 dilutions) of each phage in sterile SM buffer, 100 µL of the phage lysate was added to 200 µL of *E. coli *WG5 DSM 18455 (host bacteria) culture grown for 24 hours in LB broth at 37 °C with shaking at 100 rpm. Then, 5 mL of molten LB agar (0.7% W/V) was added, mixed gently, and poured on sterile LB agar plates, left to dry for 10 minutes, and it was incubated at 37 °C for 24 hours. This test was run in triplicate for each phage isolated. After incubation, the plaques formed on the plates, and the phage titer was calculated as PFU/ML = Plaques per plate X dilution factor / Volume of phage plated in mL.

Phage lysis profile determination

A phage host profile was carried out using a spot test assay to check if the isolated phages had the ability to lyse other organisms. Lytic spectra of a phage mean the range of organisms (bacteria) that a phage can kill. The spot assay was based on the ability of the phages to either lyse (plaque production) or not lyse (no plaque production) [6] against eight selected bacterial isolates (*S. typhimurium *ATCC14028, *S. typhimurium *WG49 NCTC 12484, *M. morganii, *ESBL *E. coli, P. mirabilis, C. freundii, Corynebacterium* spp., and *S. marcescens*). These selected bacterial isolates were first subcultured in LB broth, apart from *Corynebacterium* spp., which were subcultured in trypticase soy broth and were incubated at 37 °C for 24 hours, with shaking at 100 rpm. Plates were prepared and marked for each phage lysate isolated. After 24 hours of growth at an OD of 0.5 at 600 nm, 200 µL of this grown bacterial culture was picked and mixed with 5 mL of soft agar, poured onto the LB agar plates, and left for 10 minutes to solidify. After, 10 µL droplets of each phage lysate were spotted on LB agar plates (with the test bacteria) using sterile pipettes. The plates were left to dry for five minutes and incubated at 37 °C for 24 hours and then checked for lysis zones [10].

Temperature stability test

Plaque-forming units were used to measure changes in phage survival against different temperatures (T°). The isolated phages were subjected to -20 °C (freezing T°), 4 °C (refrigeration T°), 25 °C (room T°), 37 °C (body T°), and 80 °C (stress T°). After incubation at the respective temperature for 20 minutes, the phage survival was evaluated using the double agar layer method as previously explained.

Phage dilution endpoint (DEP)

The DEP of the isolated phages was determined by the spot test. The DEP was done on dilutions from 10-^1^ to 10-^12^. Plates were prepared and marked for each phage and dilution. The host bacterium (*E. coli *WG5 DSM 18455) was grown until the culture reached an OD of 0.5 at 600 nm), and 200 µL of host bacteria was mixed with 5 mL molten soft LB agar (0.7% W/V), poured onto LB agar plates, and allowed to solidify for 10 minutes. After, 10 µL droplets of each phage lysate dilution were spotted on LB agar plates using sterile pipettes. The plates were left to dry for five minutes and incubated at 37 °C for 24 hours and then checked for zones of lysis [10].

Data management and analysis

The data were summarized in percentages/proportions and frequencies and presented in graphs and tables. GraphPad Prism version 8 (GraphPad Software, Inc.) was used.

## Results

Bacteriophage isolation

**Figure 1:**
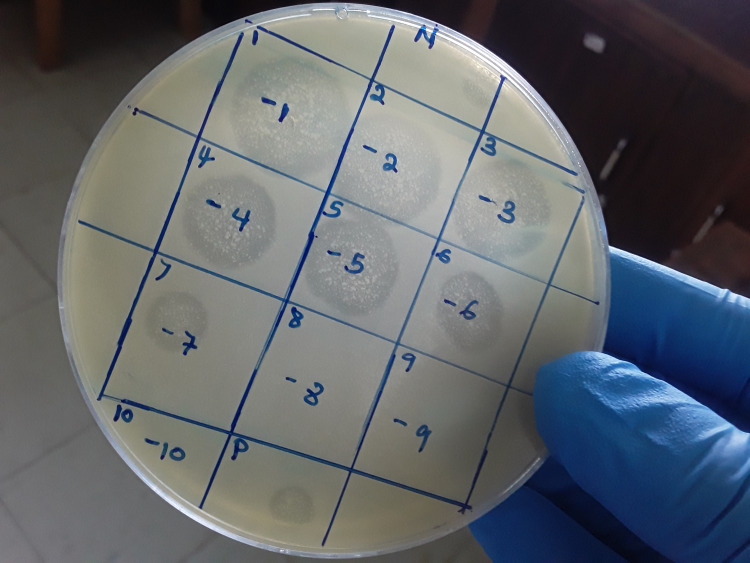
Showing zones of bacteriophage lysis during the spot test to determine the phage lytic activity During the spot test to determine the phage lytic activity N - Negative control, P - positive control, number 1-10 - label of phage dilution, 10^-1^ to 10^-10^ - phage dilutions

In total, 17 samples were screened for the presence of phages. Using one bacterial host strain (*E. coli *WG5 DSM 18455), 17 phages were isolated from sewage water, hospital waste, and river water by the double agar overlay method (DLA). The phages lytic to the host bacterium were isolated from all the samples. These phages produced clear and turbid plaques of varying sizes (Figure 1). Results were denoted positive if there was a clear zone of lysis (clear plaques) on the agar plates. The isolated phages were named according to the host organism (*E. coli *WG5 DSM 18455), sample collection site, person who isolated them, and date of isolation (e.g., ECWG5_H_OJ_25). The spectrum of phages isolated against the host bacterium is illustrated in Table 2. The plaque-forming unit per mL (Pfu/mL) was calculated as the number of plaques formed divided by the volume of phage used multiplied by the dilution factor.

**Table 2:** Phage lytic profile +- Lytic activity present, - Lytic activity absent EC - *Escherichia coli*, E - effluent, I - influent, H - hospital, DS - downstream, US - upstream, RRwi - River Rwizi, Mbra - Mbarara, Kle - Kabale, Kis - Kisoro, Ntu - Ntungamo, RKir - River Kiruruma, Kig - Kigongi, Kiz - Kizungu, Kat - Katete, Kak - Kakoba, OJ - initials of person who isolated the phages, 25 - year phages were isolated, ATCC - American-type culture collection

Phage	WG49 *Salmonella typhimurium*	*Salmonella typhimurium *ATCC14028	ESBL *E. coli*	Morganelli morganii	Proteus mirabilis	Citrobacter freundii	*Corynebacterium *spp.	Serratia marcescens	Total bacteria lysed
ECWG5_H_OJ_25	-	-	-	-	+	-	+	-	2
ECWG5_RRwiDS_OJ_25	-	-	-	-	+	-	-	-	1
ECWG5_RRwiUS_OJ-25	-	-	-	-	+	-	-	-	1
ECWG5_I_MbraKiz_OJ_25	+	+	+	-	+	-	-	-	4
ECWG5_E_MbraKiz_OJ_25	-	-	+	-	+	-	-	-	2
ECWG5_I_MbraKat_OJ_25	+	+	+	-	+	+	+	-	6
ECWG5_E_MbraKat_OJ_25	+	+	+	-	+	+	-	-	5
ECWG5_I_MbraKak_OJ_25	+	+	-	-	+	-	-	-	3
ECWG5_E_MbraKak_OJ_25	-	+	-	-	+	-	-	-	2
ECWG5_RKirDS_OJ_25	-	-	-	-	+	-	+	-	2
ECWG5_RKirUS_OJ_25	+	+	+	+	-	+	-	+	6
ECWG5_I_KleKig_OJ_25	+	+	+	+	-	+	+	+	7
ECWG5_E_KleKig_OJ_25	+	-	-	-	+	-	-	+	3
ECWG5_I_Ntu_OJ_25	+	+	+	+	-	+	+	+	7
ECWG5_E_Ntu_OJ_25	+	+	-	+	-	-	-	-	3
ECWG5_I_Kis_OJ_25	+	+	+	+	-	+	+	+	7
ECWG5_E_Kis_OJ_25	+	+	+	+	-	+	+	+	7
Total phase lysing bacteria	11	11	9	6	11	7	7	6	

Phage Lytic Determination

The phage lytic was determined by cross-infection between the isolated phages and host bacteria by spot test assay. The infectivity of 17 phages isolated was tested against eight selected bacterial clinical isolates, including *S. typhimurium *ATCC14028,* S. typhimurium *WG49 NCTC 12484,* M. morganii, *ESBL *E. coli, P. mirabilis, C. freundii, Corynebacteria *spp., and *S. marcescens*. Most of the phages were able to lyse, as shown in Figure 1, at least two of the bacterial species tested. The infectivity of the different phages was grouped as lytic activity present (+) and lytic activity absent (-) (Table 2).

**Figure 2:**
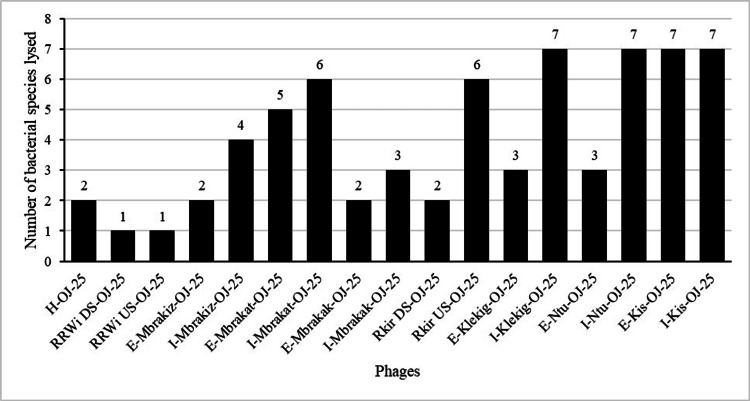
Phage lytic activity against the number of bacterial strains lysed H - Hospital, RRWI - River Rwizi, DS - downstream, E - effluent, Mbrakiz - Mbarara Kijungu, I - influent, Mbrakat - Mbarara Katete, Mbrakak - Mbarara Kakoba, RKir - River Kiruruma, US - upstream, Klekig - Kabale Kigongi, Ntu - Ntungamo, Kis - Kisoro, OJ - initials of person who isolated phages, 25 - year of phage isolation

**Figure 3:**
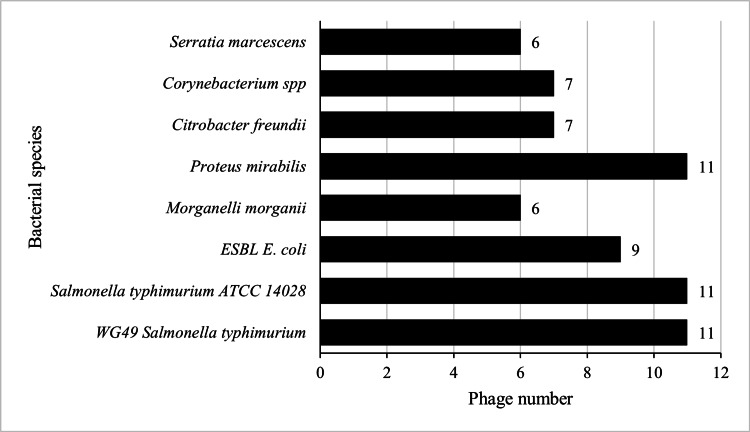
Phage number against bacterial species lysed The phages were most active against *Salmonella *spp. *and Proteus mirabilis *(11/17), followed by ESBL *E. coli* (9/17), and the least lystic activity was against *Morganelli morganii* and *Serratia marcescens* (6/17).

Among the 17 phages, ECWG5_I_KleKig_OJ_25, ECWG5_I_Ntu_OJ_25, ECWG5_I_Kis_OJ_25, and ECWG5_I_Kis_OJ_25 were the most effective phages with 88% lytic ability against seven bacterial species each (Figure 2). More than half of the phages (nine out of 17) were found to lyse the ESBL *E. coli *strain (Figure 3).

Phage temperature stability test

Table 3 summarizes the effect of temperature on the phages isolated. All phages isolated survived at 4 °C, followed by (16/17) at 37 °C, 15/17 at -20 °C, and 12/17 at 25 °C. Only three out of the 17 isolated phages (*ECWG5_RRwiDS_OJ_25, ECWG5 _I_MbraKiz_OJ_25,* and *ECWG5_I_MbraKat_OJ_25)* survived at 80 °C.

**Table 3:** Effect of different temperatures on phage survival °C - Centigrade degrees Celsius, PFU/mL - plaque forming unit per mL, EC - *Escherichia coli*, E - effluent, I- influent, H - hospital, DS - downstream, US - upstream, RRwi - River Rwizi, Mbra - Mbarara, Kle - Kabale, Kis - Kisoro, Ntu - Ntungamo, RKir - River Kiruruma, Kig - Kigongi, Kiz - Kizungu, Kat - Katete, Kak - Kakoba, OJ - initials of person who isolated the phages, 25 - year phages were isolated

Phage	Temperature (°C)/phage titer in PFU/mL				
	-20	4	25	37	80
ECWG5_H_OJ_25	6 x 10^10^	7.7 x 10^10^	2.1 x 10^10^	5.8 x 10^10^	0
ECWG5_RRwiDS_OJ_25	2.54 x 10^8^	3.0 x 10^8^	4.3 x 10^7^	2.04 x 10^8^	2.3 x 10^7^
ECWG5_RRwiUS_OJ-25	2.26 x 10^8^	6.3 x 10^7^	6 x 10^6^	1.72 x 10^8^	0
ECWG5_I_MbraKiz_OJ_25	2.56 x 10^8^	9.2 x 10^7^	1.4 x 10^7^	1.68 x 10^8^	4 x 10^7^
ECWG5_E_MbraKiz_OJ_25	2 x 10^8^	2.68 x 10^8^	0	2.56 x 10^8^	0
ECWG5_I_MbraKat_OJ_25	0	3.3 x 10^7^	9 x 10^6^	2.1 x 10^7^	5 x 10^6^
ECWG5_E_MbraKat_OJ_25	1.5 x 10^7^	1.46 x 10^8^	0	0	0
ECWG5_I_MbraKak_OJ_25	2.66 x 10^8^	2.6 x 10^8^	1.2 x 10^7^	2.4 x 10^8^	0
ECWG5_E_MbraKak_OJ_25	4.7 x 10^7^	2.49 x 10^8^	2.3 x 10^7^	4.7 x 10^7^	0
ECWG5_RKirDS_OJ_25	3.6 x 10^10^	5.1 x 10^10^	5.1 x 10^10^	2.17 x 10^11^	0
ECWG5_RKirUS_OJ_25	0	1.18 x 10^12^	0	2.07 x 10^12^	0
ECWG5_I_KleKig_OJ_25	6.1 x 10^11^	8.5 x 10^11^	3.3 x 10^11^	2.86 x 10^12^	0
ECWG5_E_KleKig_OJ_25	4.2 x 10^10^	1.4 x 10^11^	0	1.76 x 10^11^	0
ECWG5_I_Ntu_OJ_25	1.49 x 10^12^	1.94 x 10^12^	2.6 x 10^11^	1.8 x 10^12^	0
ECWG5_E_Ntu_OJ_25	1.25 x 10^11^	1.69 x 10^11^	2.4 x 10^10^	2.11 x 10^11^	0
ECWG5_I_Kis_OJ_25	5.3 x 10^10^	1.09 x 10^11^	0	2.16 x 10^11^	0
ECWG5_E_Kis_OJ_25	1.4 x 10^12^	1.1 x 10^12^	1.8 x 10^11^	1.58 x 10^12^	0

Dilution end point (DEP)

The spot test assay was carried out at serial dilutions from 10^-1^ to 10^-12^ to determine DEP of the phages and assess the highest phage dilution that showed lysis. There was variation in DEP per the different phages isolated, as summarized in Table 4. The DEP of the isolated phages ranged from 10^-6^ to 10^-10^. Four phages showed the highest DEP of 10^-10^, while the majority of the phages (11/17) had a DEP of 10^-8 ^or below.

**Table 4:** Dilution end point of isolated phages +- Lysis, - No lysis EC - *Escherichia coli*, E - effluent, I - influent, H - hospital, DS - downstream, US - upstream, RRwi - River Rwizi, Mbra - Mbarara, Kle - Kabale, Kis - Kisoro, Ntu - Ntungamo, RKir - River Kiruruma, Kig - Kigongi, Kiz - Kizungu, Kat - Katete, Kak - Kakoba, OJ - initials of person who isolated the phages, 25 - year phages were isolated

Phage	Dilution endpoint											
	10^-1^	10^-2^	10^-3^	10^-4^	10^-5^	10^-6^	10^-7^	10^-8^	10^-9^	10^-10^	10^-11^	10^-12^
ECWG5_H_OJ_25	+	+	+	+	+	+	+	+	-	-	-	-
ECWG5_RRwiDS_OJ_25	+	+	+	+	+	+	+	+	-	-	-	-
ECWG5_RRwiUS_OJ-25	+	+	+	+	+	+	-	-	-	-	-	-
ECWG5 _I_MbraKiz_OJ_25	+	+	+	+	+	+	+	+	-	-	-	-
ECWG5_E_MbraKiz_OJ_25	+	+	+	+	+	+	+	+	-	-	-	-
ECWG5_I_MbraKat_OJ_25	+	+	+	+	+	+	+	+	-	-	-	-
ECWG5_E_MbraKat_OJ_25	+	+	+	+	+	+	-	-	-	-	-	-
ECWG5_I_MbraKak_OJ_25	+	+	+	+	+	+	+	+	-	-	-	-
ECWG5_E_MbraKak_OJ_25	+	+	+	+	+	+	+	+	-	-	-	-
ECWG5_RKirDS_OJ_25	+	+	+	+	+	+	+	+	-	-	-	-
ECWG5_RKirUS_OJ_25	+	+	+	+	+	+	+	+	+	+	-	-
ECWG5_I_KleKig_OJ_25	+	+	+	+	+	+	+	+	+	+	-	-
ECWG5_E_KleKig_OJ_25	+	+	+	+	+	+	+	+	-	-	-	-
ECWG5_I_Ntu_OJ_25	+	+	+	+	+	+	+	+	+	+	-	-
ECWG5_E_Ntu_OJ_25	+	+	+	+	+	+	+	+	-	-	-	-
ECWG5_I_Kis_OJ_25	+	+	+	+	+	+	+	+	-	-	-	-
ECWG5_E_Kis_OJ_25	+	+	+	+	+	+	+	+	+	-	-	-

## Discussion

The study aimed at isolating phages and determining their lytic profile from the river and wastewater in south-western Uganda. We isolated 17 phages using *E. coli* WG5 DSM 18455 as a host, a finding that shows that phages are highly abundant in aquatic ecosystems, including rivers, water ponds, and freshwater bodies, where they interact with local bacterial populations [13-15]. Phages have also been isolated successfully from poultry/animal waste [16]. Moreover, Alonso et al. isolated 26 phages from water samples of the Alboran Sea [17]. In addition, other researchers isolated phages from soil [18]. This indicates that phages can be isolated from diverse sources.

Our study isolated phages that formed plaques, confirming that the bacteria in that specific area were susceptible to the phage's infection. These phages appeared to have lytic activity as observed by clear plaques. Lytic phages are recommended to control bacterial populations because of their ability to immediately kill their hosts without incorporating their genome into the infected host cell [19]. Moreover, we isolated phages with lytic activity against Gram-positive (*Corynbacterium *spp.) and Gram-negative (*S. marcescens, S. typhimurium *spp., ESBL *E. coli, M. morganii, P. mirabilis, *and *C. freundii*) bacteria. This difference could be attributed to the difference in the host organisms used (*S. aureus)*, types of samples (sewage of various livestock farms), and or geographic location (India). On the other hand, our findings are in agreement with previous researchers that isolated phages that were lytic against a variety of organisms that included Gram positives [14].

The study also determined the host range of the isolated phages. Of the 17 phages isolated, *ECWG5_I_KleKig_OJ_25, ECWG5_I_Ntu_ OJ _25, ECWG5_ I_Kis_OJ_25, *and *ECWG5_E_Kis_OJ_25* had a broad host range with 88% effectiveness against eight selected clinical bacterial strains. Hasanian et al. isolated phages against *E. coli, Salmonella *spp., *P. aeruginosa, E. cloacae, *and *M. morganii *strains [6]. There are many reports about the isolation of* E. coli, Salmonella *spp., *P. aeruginosa, E. cloacae, *and other bacterial strains' phages globally, but limited findings about *M. morganii* phages. However, findings by Hasanain et al. [6] show they isolated *M. morganii* phages. This correlates with our findings, which report 6/17 phages isolated lysed *M. morganii.* This variation in host range among phages isolated in our study compared to what other researchers isolated previously could be due to the genetic diversity of phages, hosts used, environmental niches, and phage bacterial co- evolution. In addition, isolation methods may have contributed to the isolation of phages specific to certain bacteria [20,21].

The study results indicate that isolated phages exhibit varying degrees of host specificity, an indication that the phages do not universally infect every bacterial strain present. It may be attributed to the fact that phages differ in morphological structures, host receptors, and defense mechanisms to attack different bacterial host species [14].

We also isolated phages that were found to be effective against ESBL *E. coli* (9/17). These findings show potential use of phages in managing ESBL *E. coli*-related infections. Our findings are similar to those of other studies that have isolated phages against various organisms, including resistant strains [19,22].

Temperature is crucial for stabilizing phage proteins [5]. In the present study, most of the phages (14/17) became inactivated at 80 °C. Our findings are similar to those of Rattanachaikunsopon et al., who reported that phages can be inactivated at 80 °C [19]. On the contrary, Hasanain et al. revealed that* E. coli, P. aeruginosa*, and *Salmonella *spp. phages were able to survive at 70 °C and 80 °C, and their thermal inactivation point was at 90 °C [6]. This variation in findings could be due to the different host bacteriaused to isolate the phages and bacterial evolution over time.

The study isolated 17 phages from wastewater and river water, which showed lytic activity against selected clinical Gram-positive and Gram-negative bacterial isolates. This study also provides insights into phage survivability under different temperature conditions. In addition, the study revealed that some isolated phages maintained lytic activity at relatively high dilutions (10^-10^). This work provides preliminary information about the isolated phages for researchers to further characterize these phages to determine their potential use in clinical applications of phage-based interventions, in particular in the management of drug-resistant pathogenic bacteria.

Study limitation

This study did not examine the phages' physical characteristics using essential techniques such as transmission electron microscopy (TEM). This leaves their structural type and classification uncertain. In addition, the study lacked genomic characterization of the isolated phages, so the absence of screening for genes associated with lysogeny, virulence, or antibiotic resistance means that their safety for potential therapeutic use remains unconfirmed. In addition, the phages' lytic capacity was assessed against only a narrow selection of bacterial strains. A broader evaluation using a diverse panel of clinical isolates would have better defined their potential scope of treatable infections and utility for broad-spectrum application.

Recommendations

Successful phage therapy requires isolating broad-host or strain-specific phages from environmental sources, such as soil, that can effectively target pathogenic and antibiotic-resistant bacteria. To achieve this, researchers must utilize a diverse array of bacterial strains to fully map phage-host specificity and infection dynamics. Furthermore, integrating whole-genome sequencing with in vitro and in vivo preclinical studies is essential for identifying potential virulence or antibiotic-resistance genes, ensuring the selection of safe therapeutic candidates. Understanding phage stability also necessitates investigating their survival across various chemical environments and pH levels beyond just temperature. Finally, utilizing TEM is vital for characterizing phage morphology, providing detailed data on their size, shape, and structural features.

## Conclusions

The study isolated 17 phages from wastewater and river water, which showed lytic activity against selected clinical Gram-positive and Gram-negative bacterial isolates. This study also provides insights into phage survivability under different temperature conditions. In addition, the study revealed that some isolated phages maintained lytic activity at relatively high dilutions (10^-10^). This work provides preliminary information about the isolated phages for researchers to further characterize these phages to determine their potential use in clinical applications of phage-based interventions, in particular in the management of drug-resistant pathogenic bacteria.
